# Prevalence of *Coxiella burnetii* in German sheep flocks and evaluation of a novel approach to detect an infection via preputial swabs at herd-level

**DOI:** 10.1017/S0950268820000679

**Published:** 2020-03-16

**Authors:** A. Wolf, T. L. Prüfer, C. Schoneberg, A. Campe, M. Runge, M. Ganter, B. U. Bauer

**Affiliations:** 1 Clinic for Swine, Small Ruminants and Forensic Medicine, University of Veterinary Medicine Hannover, Foundation, Hannover, Germany; 2 Lower Saxony State Office for Consumer Protection and Food Safety (LAVES), Food and Veterinary Institute Braunschweig/Hannover, Hannover, Germany; 3 Department of Biometry, Epidemiology and Information Processing, (IBEI), WHO Collaborating Centre for Research and Training for Health at the Human-Animal-Environment Interface, University of Veterinary Medicine Hannover, Foundation, Hannover, Germany

**Keywords:** *Coxiella burnetii*, small ruminants, surveillance, venereal transmission

## Abstract

A prevalence study was conducted on German sheep flocks including goats if they cohabitated with sheep. In addition, a novel approach was applied to identify an infection at the herd-level before lambing season with preputial swabs, suspecting venereal transmission and ensuing colonisation of preputial mucosa with *Coxiella (C.) burnetii*. Blood samples and genital swabs were collected from breeding males and females after the mating season and were analysed by enzyme-linked immunosorbent assay (ELISA) and quantitative polymerase chain reaction (qPCR) respectively. In total, 3367 animals were sampled across 71 flocks. The true herd-level prevalence adjusted for misclassification probabilities of the applied diagnostic tests using the Rogan-Gladen estimator for the prevalence estimate and a formula by Lang and Reiczigel (2014) for the confidence limits, ranged between 31.3% and 33% (95% confidence interval [95% CI] 17.3–45.5) detected by the ELISA and/or qPCR. Overall 26–36.6% (95% CI 13–56.8) were detected by ELISA, 13.9% (95% CI 4.5–23.2) by the qPCR and 7.9–11.2% (95% CI 0.08–22.3) by both tests simultaneously. The range of results is due to data obtained from literature with different specifications for test quality for ELISA. Among eight farms with females shedding *C. burnetii,* three farms (37.5%) could also be identified by preputial swabs from breeding sires. This indicates less reliability of preputial swabs if used as a single diagnostic tool to detect *C. burnetii* infection at the herd-level.

## Introduction

Q fever is a zoonotic disease caused by the intracellular bacterium *Coxiella burnetii*. In many countries, ruminants are the main source of human epidemics [1]. However, other animals like cats and dogs can also be responsible for human Q fever infections [2, 3]. Infected females can shed large amounts of the pathogen into the environment during abortion or even upon normal delivery through birth products. Moreover, the bacterium is also shed with milk, faeces and urine [4]. In animals and humans alike, the main infection pathway is the inhalation of pathogen-contaminated aerosols [5]. Aside from airborne dissemination of *C. burnetii*, sexual transmission has occasionally been reported in humans [6]. Furthermore, transmission of the pathogen via sexual intercourse was demonstrated in mice [7]. Later on, *C. burnetii* was also detected in rams` semen [8]. Hence, in sheep, transmission during breeding could result either from direct contact between mucosa of the reproductive organs of rams and ewes or from semen containing *C. burnetii*. Different pathogens like *Chlamydia* spp. and *Brucella* spp. were detected in samples taken from the preputial mucosa in rams [9, 10]. But this sampling method has rarely been used to diagnose *C. burnetii* infection in small ruminants [11].

Germany has a long history of *C. burnetii* infections. In 1947, the pathogen was first diagnosed within the context of a human outbreak in the federal state of Baden-Wuerttemberg [12]. Most human Q fever cases occurred in southern Germany, but reports from central and northern regions increased with occasional larger outbreaks and mainly associated with lambing sheep [13–15]. Despite the awareness of *C. burnetii* infection in the German sheep population, only poor data, especially in the northern federal states, are available. Therefore, the first aim of this study was to assess the actual prevalence of *C. burnetii* in German sheep flocks. For this purpose, a cross-sectional study encompassing five federal states with high numbers of sheep was conducted. In addition, we hypothesised that transmission of *C. burnetii* occurs during sexual intercourse of small ruminants and that the pathogen may be localised in the prepuce of infected rams. Therefore, preputial swabs of breeding sires were examined after the mating season in order to substantiate this assumption. As a consequence of *C. burnetii* recognition at an early stage of infection, measures such as pre-lambing treatments could be performed in order to reduce abortion and shedding of the pathogen to minimise the risk for human infection. Overall, conclusive results could contribute to develop a monitoring and surveillance system (MOSS) identifying *C. burnetii* in small ruminant flocks.

## Material and methods

### Animals and sample collection

Basic data on small ruminant populations in Germany were sourced from the Genesis-Online Database of the German Federal Statistical Office from 1st March 2016 and used to calculate the number of samples [16]. Applied statistical methods for calculating the number of samples were performed according to the STROBE Statement below [17]. Due to reasons of time and costs a maximum of 71 herds could be investigated in the five selected federal states during the study. Considering a herd-level prevalence of 10% [18] and a 95% confidence interval, this resulted in a maximum precision of ±7% [PASS 16 Power Analysis and Sample Size Software (2018). NCSS, LLC. Kaysville, Utah, USA, ncss.com/software/pass].

To represent the distribution of sheep farms between the federal states, the following number of farms (calculated proportion of the federal states in the sample according to the Genesis-Online Database of the German Federal Statistical Office from 1st March 2016 [16]) were included into the study: Schleswig-Holstein 12 (16.7%), Lower Saxony 11 (13.6%), North Rhine-Westphalia 12 (16.7%), Baden-Wuerttemberg 14 (19.7%) and Bavaria 22 (33.3%). These states have the largest sheep populations within Germany. Therefore, the estimation of *C. burnetii* occurrence in these states is important for further risk assessments. Moreover, other federal states have smaller numbers of sheep and especially in eastern parts of Germany, examinations concerning *C. burnetii* infection in sheep have been conducted in recent years [19, 20]. The participating farms were selected based on the respective owners' willingness to contribute to the study. Hence, this study was based on a convenient sampling.

The number of samples required from each flock to estimate the positivity rate independently of a clinical disease was calculated on the assumption of 3% expected prevalence [18], 95% confidence interval, 80% power and 5% precision. A maximum of 44 animals per herd had to be sampled. If goats were kept on the same farm, their sample size was calculated under the same assumptions, independently of the number of sheep sampled. Genital swabs (Sarstedt, Nürmbrecht, Germany) from breeding males and females in combination with blood samples (Kabe Labortechnik, Nürmbrecht-Elsenroth, Germany) were collected. Blood samples were centrifuged within 6 h of sampling. The genital swabs and serum samples were stored at −18 °C until laboratory examination. Aside from their species, the sex and the reproductive status of the females (gimmer or adult ewe) were recorded. The flocks were visited between November 2017 and June 2018, after or during the mating season when breeding sires were introduced into the flock for at least 6 weeks. Farms vaccinating against *C. burnetii* were excluded.

### Detection methods

*C. burnetii*-specific DNA in the genital swabs was detected by amplificating IS*1111* elements with a quantitative polymerase chain reaction (qPCR) (LSI VetMAX™*Coxiella burnetii*, Thermo Fisher Scientific, Germany). The manufacturer indicates Cycle Threshold (*C*_t_) values ⩽ 45 as positive and *C*_t_ values > 45 as negative. A sensitivity of 95–100% and a specificity of 100% were assumed according to the manufacturers validation report from 8th December 2014.

The *C. burnetii*-specific antibody levels in the serum samples were determined by enzyme-linked immunosorbent assay (ELISA), detecting phase unspecific IgG antibodies (Q Fever Antibody Test Kit, IDEXX, Switzerland). The manufacturers specified samples with S/P (%) > 40 as positive, values with a percentage <30 as negative. Results with values between 30% and 40% were considered inconclusive and were scored as negative in the present study. A sensitivity of 70.1%, 84% and 98.6% and a specificity of 96.2%, 99% and 97.1% were assumed according to Muleme *et al*., Paul *et al*. and Horigan *et al*. [21–23], taking into account that data were provided under different study designs and for different species.

### Statistical analysis

#### Occurrence of *C. burnetii* in German sheep flocks

Both diagnostic tests (ELISA and qPCR) were considered for the estimation of herd-level prevalence and the proportion of *C. burnetii* infected adults within the farms. The results were divided into the following test combinations: ELISA+, qPCR+, ELISA and qPCR+, ELISA and/or qPCR+. After sampling on the selected farms, the selections were checked to determine whether the sample survey was representative. For all calculations, we used the statistical software SAS (SAS Institute Inc., Cary, NC, USA).

#### Herd-level prevalence

A farm was considered positive if at least one sampled animal yielded a positive test result. The herd-level prevalence was assessed across the five selected federal states. To determine the true herd-level prevalence, the apparent prevalence was corrected for misclassification probabilities (sensitivity and specificity of the diagnostic tests) using the Rogan-Gladen estimator [24] for the prevalence estimate and a formula by Lang and Reiczigel for the confidence limits [25]. Although not substantiated by the sample size, the results were analysed for a possible impact of federal state and management systems of the farm on the occurrence of *C. burnetii*.

#### Proportion of *C. burnetii* infected adults within the farms

An animal was considered positive if at least qPCR or ELISA presented a positive test result. The results for the proportion of infected adults within the farms were corrected for misclassification probabilities as well.

## Results

### Occurrence of *C. burnetii* in German sheep flocks

**Fig. 1.**
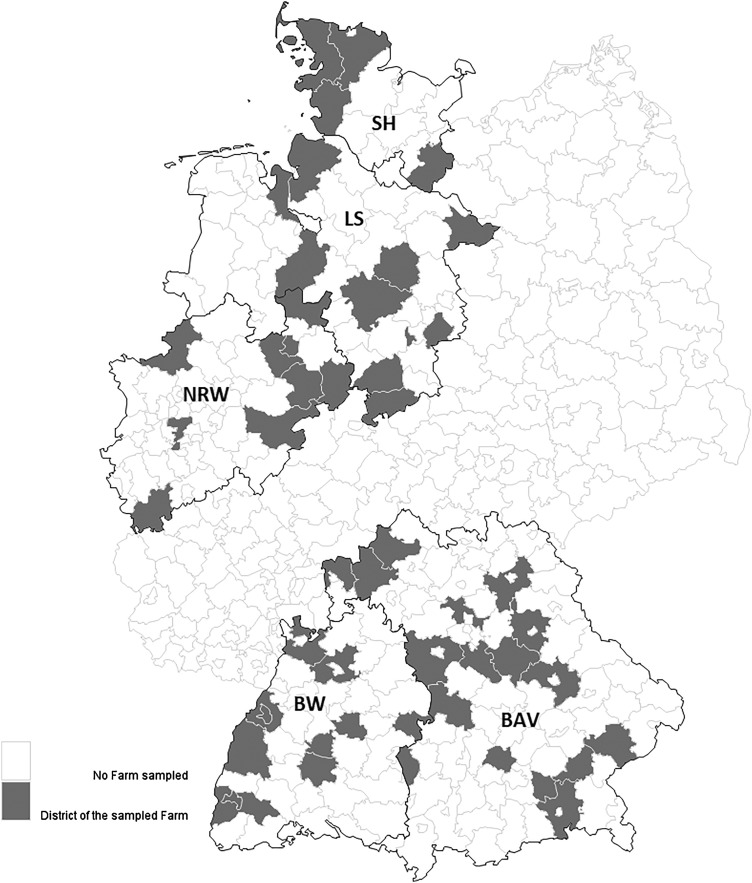
Distribution of the 71 sampled farms in the five selected German federal states. Districts with grey background indicating location of the participating farms. Federal states: SH = Schleswig-Holstein; LS = Lower Saxony; NRW = North Rhine-Westphalia; BAV = Bavaria; BW = Baden-Wuerttemberg.

Samples from 3367 animals (2920 sheep and 447 goats) belonging to 71 flocks (41 sheep and 30 mixed flocks) were examined (Fig. 1). The included federal states have the highest number of sheep within Germany and represent 61.3% (1 123 877/1 834 275) of the entire German sheep population [16]. The distribution of the examined farms in the federal states was compared to calculated data sourced from the Federal Statistical Office for total numbers of farms in each federal state. Bavaria is slightly underrepresented (31%) and Lower Saxony overrepresented (15.5%). In addition, we verified whether the ratio of pure sheep farms (57.8%) to mixed sheep and goat farms (42.3%) in the present survey corresponds to the actual distribution within federal states. The proportion of mixed farms is overrepresented comparing the data from the State Statistical Office of Lower Saxony for sheep farms (80.9%) and for mixed farms (19.1%) [26]. This bias must be taken into account when interpreting the results.

### Herd-level prevalence

Overall, the apparent prevalence of *C. burnetii* infections (ELISA and/or qPCR) at the herd-level was 33.8% (*n* = 24) (Table 1). The true herd-level prevalence adjusted for misclassification probabilities of the applied diagnostic tests using the Rogan-Gladen estimator for the prevalence estimate [24] and a formula by Lang and Reiczigel for the confidence limits [25] is presented in Table 2. Assuming the test quality according to Muleme *et al*. [21], the true herd-level prevalence was 31.3%, according to Paul *et al*. [22] or Horigan *et al*. [23], the true prevalence results in 33% and 31.4% respectively.

**Table 1.** Percentage of *C. burnetii* positive farms by federal state and apparent herd-level prevalence of all examined farms (for four different definitions of positive herd status)

Federal state	Number of farms sampled *n*	ELISA positive farms *n* (%)	qPCR positive farms *n* (%)	ELISA and qPCR positive farms *n* (%)	ELISA and/or qPCR positive farms *n* (%)
Percentage and number of farms tested positive per federal state					
SH	12	2 (16.7)	–	–	2 (16.7)
LS	11	1 (9.1)	1 (9.1)	–	2 (18.2)
NRW	12	2 (16.7)	–	–	2 (16.7)
BAV	22	6 (27.3)	4 (18.2)	3 (13.6)	7 (31.8)
BW	14	9 (64.3)	5 (35.7)	3 (21.4)	11 (78.6)
Apparent herd-level prevalence					
Total	71	20 (28.2)	10 (14.1)	6 (8.5)	24 (33.8)

Federal states: SH = Schleswig-Holstein; LS = Lower Saxony; NRW = North Rhine-Westphalia; BAV = Bavaria; BW = Baden-Wuerttemberg.

**Table 2.** True herd-level prevalence of all examined farms adjusted for sensitivity and specificity of the applied test systems ELISA and qPCR (for four different definitions of positive herd status) according to the literature with reliable estimation for test quality for ELISA and the manufacturer`s validation report for the qPCR

Diagnostic test (reference for test quality)	Sensitivity (%)	Specificity (%)	True herd-level prevalence (confidence interval) (%)
ELISA (Muleme *et al.* [21])	70.1	96.2	36.6 (16.4–56.8)
ELISA (Paul *et al.* [22])	84	99	32.5 (17.9–47)
ELISA (Horigan *et al.* [23])	98.6	97.1	26 (13–38.9)
qPCR (Validation Report 2014, LSI™)	95	100	13.9 (4.5–23.2)
ELISA and qPCR (Muleme *et al.* [21]/LSI™)	66.6	100	11.2 (0.1–22.3)
ELISA and qPCR (Paul *et al.* [22]/LSI™)	79.8	100	9.3 (0.1–18.5)
ELISA and qPCR (Horigan *et al.* [23]/LSI™)	93.2	100	7.9 (0.08–15.7)
ELISA and/or qPCR (Muleme *et al.* [21]/LSI™)	98.5	96.2	31.3 (17.3–45.2)
ELISA and/or qPCR (Paul *et al.* [22]/LSI™)	99.2	99	33 (20.5–45.5)
ELISA and/or qPCR (Horigan *et al.* [23]/LSI™)	99.9	97.1	31.4 (18.1–44.7)

#### Federal states

The southern states Bavaria (31.8%; 7/22) and Baden-Wuerttemberg (78.6%; 11/14) had the highest proportion of positive farms with Baden-Wuerttemberg on the top of all states (Table 1). Overall, positive farms were detected more by serology (83.3%; 20/24) than with the qPCR technique (41.7%; 10/24).

#### Flock type

**Fig. 2.**
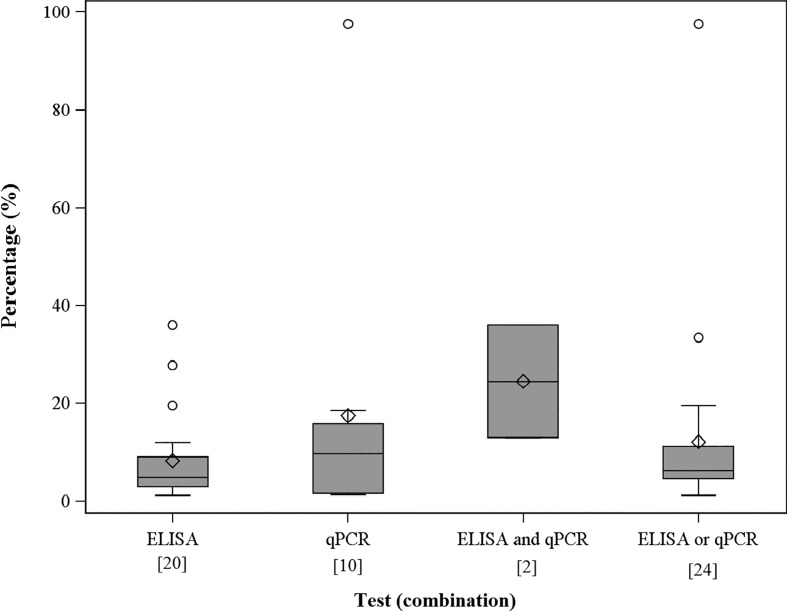
Numbers of *C. burnetii* positive and negative farms by farm type (farms keeping only sheep and farms keeping sheep and goats). Federal states: SH = Schleswig-Holstein; LS = Lower Saxony; NRW = North Rhine-Westphalia; BAV = Bavaria; BW = Baden-Wuerttemberg.

The proportion of mixed flocks (sheep and goats) and sheep flocks within positive farms is shown in Figure 2.

### Proportion of *C. burnetii* infected adults within positive farms

**Fig. 3.**
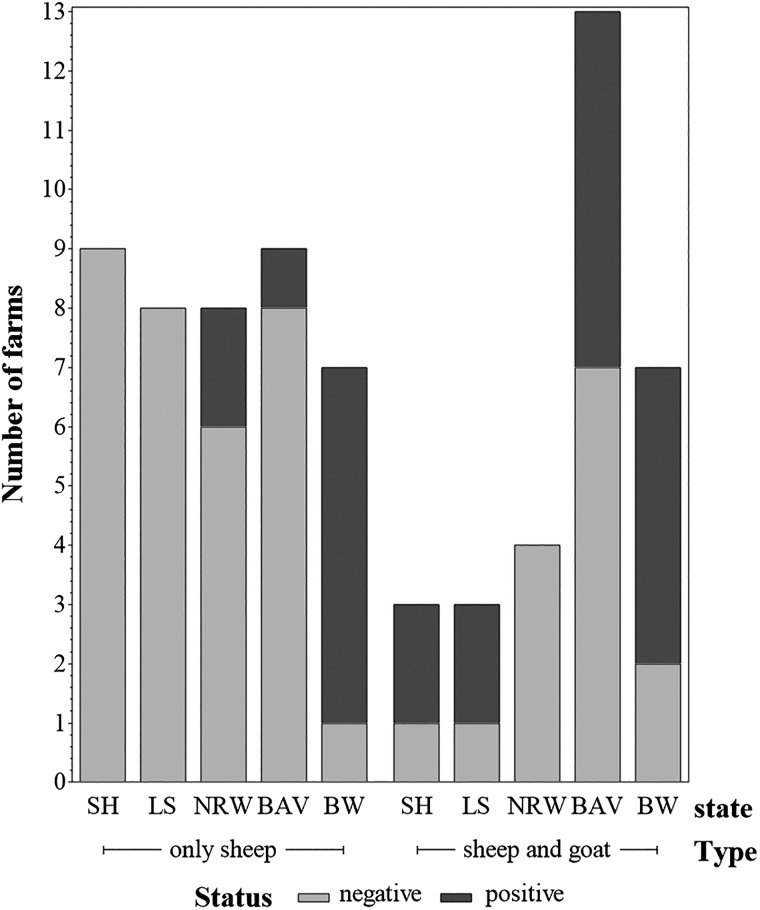
The apparent proportion of *C. burnetii* infected adults within the positive farms. Number in brackets = farms tested positive on the individual animal level of infection status. Infection status on the individual animal level acquired by four different definitions according to PCR and ELISA test results.

The apparent proportion of *C. burnetii* infected adults within the positive farms is shown in Figure 3. The true mean proportion of infected adults detected by ELISA was 13% (95% CI 3–22) among 12 positive farms, 9% (95% CI 3–14) among 20 farms and 8% (95% CI 2–13) among 15 farms according to test quality specifications by Muleme *et al*., Paul *et al*. and Horigan *et al*. respectively [21–23]. Within 10 positive farms, 18% (95% CI 0–39) were detected by the qPCR. The mean proportion was 37% (95% CI 0–100) (Muleme *et al*.), 31% (95% CI 0–100) (Paul *et al*.) and 26% (95% CI 0–100) (Horigan *et al*.) among two positive farms detected by ELISA and qPCR simultaneously [21–23]. Overall, the true mean proportion of *C. burnetii* infected adults within the positive farms was 12% (95% CI 1–22), 11% (95% CI 3–20) and 12% (95% CI 2–22) within 19, 24 and 20 positive farms detected by the ELISA and/or qPCR.

### Evaluation of preputial swabs as detection matrix for an infection with *C. burnetii* at herd-level

*C. burnetii* was detected in preputial swabs obtained from sires of five (7%) out of 71 examined farms. This corresponds to 50% of the qPCR positive farms (*n* = 10). On these five farms, females were detected as positive either by the qPCR (vaginal swabs) (*n* = 2), or ELISA (*n* = 2), or with both detection methods (*n* = 1) (Table 3). Within three of the five positive farms, in which *C. burnetii* was detected with vaginal and preputial swabs by the qPCR simultaneously, the females presented *C*_t_values from 11 to 42 with the qPCR and the proportion of infected adults presented a range between 10.4% and 97.4% (median = 15%). The *C*_t_values and the proportion of infected adults within the five farms detected by the qPCR exclusively with vaginal swabs presented a range between 34 and 42 and 1.3% to 18.5% (median = 15.6%) respectively.

**Table 3.** Distribution of percentage and number of the 24 positive farms based on sex and test system ELISA and qPCR (for four different definitions of positive herd status)

	Male				
	ELISA positive farms *n* (%)	qPCR positive farms *n* (%)	ELISA and qPCR positive farms *n* (%)	Negative farms *n* (%)	Total *n* (%)
Female					
ELISA positive farms *n* (%)	3 (12.5)	2 (8.3)	–	10 (41.7)	15 (62.5)
qPCR positive farms *n* (%)	–	2 (8.3)	–	2 (8.3)	4 (16.7)
ELISA and qPCR positive farms *n* (%)	1 (4.2)	1 (4.2)	–	2 (8.3)	4 (16.7)
Negative farms *n* (%)	1 (4.2)	–	–	–	1 (4.2)
Total *n* (%)	5 (20.8)	5 (20.8)	–	14 (58.3)	

## Discussion

### Occurrence of *C. burnetii* in German sheep flocks

In this study, the occurrence of *C. burnetii* was determined in 71 farms located in five federal states of Germany. First of all, the results obtained depended on the selection of the farms/animals and the test systems used (ELISA and qPCR). According to Greiner and Gardener [27], the variability of the results is caused by the sampling strategy and the estimates for the diagnostic sensitivity and specificity may vary among populations and/or subpopulations of animals. The results in this study provide an estimation of the actual distribution of *C. burnetii* in small ruminant populations within the five federal states. The selection of other farms and animals may lead to different results. It has to be taken into account that we investigated a convenient sample, which was obviously biased as compared to the target population. This could be shown regarding the ratio of pure sheep and mixed farms, at least. Hence, the prevalence estimations presented here, have to be handled cautiously when generalising the results of this study. Nevertheless, this study is the first nationwide approach to estimate the herd- and intra-herd prevalence in Germany. In addition, the results depended on misclassification probabilities of the test systems (ELISA and qPCR) used. To determine the true prevalence in this particular population study, a latent class analysis could be performed. This method has the advantage of estimating the true prevalence and the misclassification errors simultaneously [28]. However, to run a latent class analysis, there are at least three different tests needed, which test the same infection status. This was not feasible in this study.

### Herd-level prevalence

In this study, most of the positive farms were only seropositive. This indicates that they had contact with *C. burnetii*, e.g. due to previous infection. The number of farms detected by the qPCR was much lower, probably because shedding of the pathogen occurred mainly during parturition and sampling took place before the lambing season started [4]. In general, shedding before parturition does not occur [29]. However, some authors postulated a possible intermitting shedding of *C. burnetii* especially during oestrus [30, 31]. This can lead to misidentification of infected animals, but this assumption has to be investigated in future studies. The smallest percentage of the examined farms revealed positive results by the ELISA and qPCR simultaneously, which showed that the presence of *C. burnetii* did not lead inevitably to the production of detectable antibody activities and at the same time seropositivity did not prove the presence of the pathogen [32]. Some females shedding *C. burnetii* never seroconvert [33].

#### Federal states

The proportion of infected farms and animals between the federal states seems to be different and some regions may be endemically infected with *C. burnetii.* This assumption would lead to a regionally adapted MOSS. Different breeding and management systems might explain the different occurrences of *C. burnetii* in distinct parts of Germany. In southern Germany, the main breed is Merino Landrace, which lambs all year-round, including periods in the summer when conditions for the survival and aerogenic transmission of *C. burnetii* are favourable. By contrast, aerogenic transmission of *C. burnetii* seems to be less likely in northern parts of Germany where sheep have a seasonal breeding behaviour and indoor lambing in spring is common due to the cold and humid weather conditions in this region. Therefore, transmission seems to be less likely in this area. Overall, risk factors should be identified in future studies to elucidate the difference of *C. burnetii* infection between northern and southern Germany.

#### Flock type

Several studies were conducted to determine the prevalence in sheep and goat flocks [19, 20]. Less attention was paid to mixed sheep and goat flocks. In the present study more mixed flocks tested positive than flocks where only sheep were kept. However, the number of mixed flocks is overrepresented in the present study and most of the mixed flocks are located in southern Germany. This area is known for their endemic *C. burnetii* infections in small ruminants [15, 34]. Nevertheless, Anastácio *et al*. [35] made a similar observation in Portugal. The seroprevalence in mixed flocks (38.5%; 95% CI 12–65) was slightly higher than in sheep flocks (37.5%; 95% CI 21–54). Later, Rizzo *et al*. [36] also reported a higher prevalence in Italian mixed flocks (48.5%; 95% CI 34.7–62.3) compared to sheep flocks (38.7%; 95% CI 25.5–51.9). Under the same management conditions, goats seem to be more susceptible for *C. burnetii*. This increases the risk for sheep situated near to goats to get infected [37].

### Proportion of infected adults within positive flocks

The proportion of adults shedding *C. burnetii* within positive flocks is higher than the proportion of seropositive animals, probably due to sampling before detectable antibodies were available during acute infection or as described above, the absence of antibodies despite the presence of the pathogen [32]. Another reason could be the detection method used in this study. The sensitivity/specificity is higher for the qPCR (95–100%/100%) in comparison with the ELISA (70.1–98.6%/96.2–99%) [21–23]. This may lead to a sensitive detection of adults shedding *C. burnetii* by the qPCR, while the detection of positive animals by ELISA may be lower due to misclassification probabilities. One farm in this study achieved a proportion of 97% infected animals within the flock and most of the animals showed positive results in ELISA and qPCR respectively. In this case, abortion and stillbirths were reported in the subsequent lambing season, which suggests an acute infection.

### Evaluation of preputial swabs as detection matrix for an infection with *C. burnetii* at herd-level

To the authors' knowledge, our study is the first attempt to monitor small ruminant flocks using preputial swabs after the mating season, in order to identify *C. burnetii* positive flocks before the main shedding at lambing occurs. Despite the shedding and detection of *C. burnetii,* which usually takes part through parturition [4], we detected females shedding *C. burnetii* before the lambing season started. This indicates that the detection of *C. burnetii* DNA before parturition is possible according to Alsaleh *et al*. [38], who detected the pathogen in flushing media from oviduct and uteri of non-pregnant goats. Furthermore, *C. burnetii* DNA was also detected in specimens from other non-pregnant animals, e.g. cats and hares [39, 40]. In this study, *C. burnetii* was found on five farms with preputial swabs by the qPCR. Interestingly, at an individual animal level, the eleven affected rams were serologically negative even in flocks with a high proportion of infected adults. In contrast, in five other flocks, six males were seropositive but the preputial swabs were negative. There seems to be no correlation between serological ELISA results and the presence of *C. burnetii* DNA on preputial mucosa. This indicates that the presence of *C. burnetii* does not lead inevitably to the production of detectable antibodies and vice versa [32]. Nevertheless, it should be noted that intra-preputial inoculation of pathogens (e.g. *Brucella ovis, Chlamydia* spp.) under experimental conditions lead to a detectable immune response [41, 42]. Moreover, these rams are probably not infected but their prepuce got contaminated with *C. burnetii* by mating infected females.

Among eight farms in which females were shedding the pathogen, three farms (37.5%) were also detected with preputial swabs by the qPCR. Within these three farms *C. burnetii* was detected by vaginal and preputial swabs by the qPCR simultaneously, the *C*_t_ values seemed to be lower and the proportion of infected adults to be higher than in those farms in which only vaginal swabs yielded positive results. Although these findings were not significant, the status of infection and the intra-herd prevalence may have an influence on the success for the detection of the pathogen with preputial swabs. The results indicate that preputial swabs are less reliable as a single detection matrix at the herd-level. However, further research is necessary to evaluate the epidemiological role of breeding sires and the applicability of preputial swabs for a MOSS at the herd-level. The question of sexual transmission of *C. burnetii* within small ruminants and the duration of colonisation on the preputial mucosa remains to be determined. In addition, the influence of duration and timing of the mating season, as well as the number of rams per ewes should be studied.

In conclusion, this study generated new data about the occurrence of *C. burnetii* in small ruminant flocks in Germany. The risk for an infection in sheep flocks may well depend on the farm location and the presence of goats on the farm. Further investigations are needed to clarify the risk factors for small ruminant flocks in Germany to acquire *C. burnetii* infection.
