# Prevalence of *Coxiella burnetii* in German sheep flocks and evaluation of a novel approach to detect an infection via preputial swabs at herd-level – ERRATUM

**DOI:** 10.1017/S0950268820000801

**Published:** 2020-04-27

**Authors:** A. Wolf, T. L. Prüfer, C. Schoneberg, A. Campe, M. Runge, M. Ganter, B. U. Bauer

During the proofing stage for the above article, Figures 2 and 3 were inadvertently switched. Cambridge University Press apologise for this error. The correct figures are given below:

**Fig. 2.**
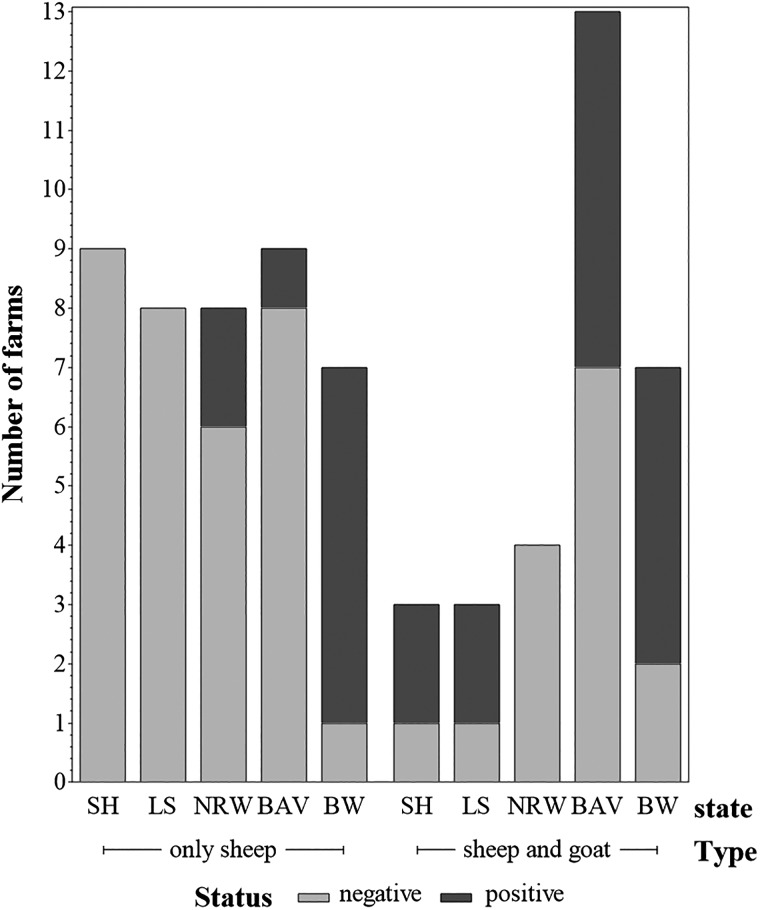
Numbers of *C. burnetii*-positive and -negative farms by farm type (farms keeping only sheep and farms keeping sheep and goats). Federal states: SH = Schleswig Holstein; LS = Lower Saxony; NRW = North Rhine-Westphalia; BAV = Bavaria; BW = Baden-Wuerttemberg.

**Fig. 3.**
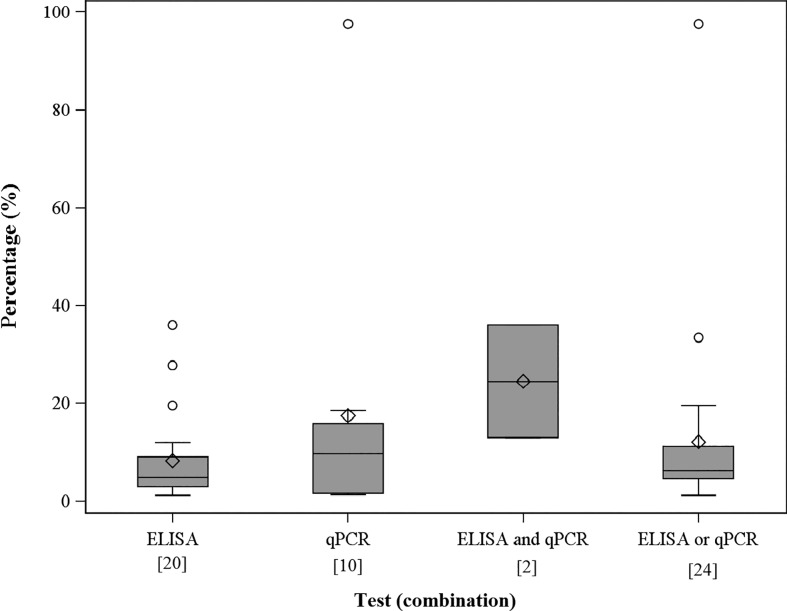
The apparent proportion of *C. burnetii*-infected adults within the positive farms. Number in brackets indicates farms tested positive on the individual animal level of infection status. Infection status on the individual animal level acquired by four different definitions according to qPCR and ELISA test results.

## References

[ref1] Wolf A, Prüfer T, Schoneberg C, Campe A, Runge M, Ganter M and Bauer B (2020) Prevalence of *Coxiella burnetii* in German sheep flocks and evaluation of a novel approach to detect an infection via preputial swabs at herd-level. Epidemiology and Infection 148, E75. 32172709 10.1017/S0950268820000679 PMC7118722

